# Clinical significance of positive fecal occult blood test in neonates

**DOI:** 10.1038/s41598-019-54511-5

**Published:** 2019-11-29

**Authors:** Qiuping Wen, Kaizhen Liu, Weihong Yue, Shiqi Shao, Shu Zhang, Xiaoqing Li, Ziyu Hua

**Affiliations:** 1 Department of Neonatology, Ministry of Education Key Laboratory of Child Development and Disorders, National Clinical Research Center for Child Health and Disorders (Chongqing), China International Science and Technology Cooperation base of Child development and Critical Disorders, Children’s Hospital of Chongqing Medical University, Chongqing, P.R. China; 2 Chongqing Key Laboratory of Child Health and Nutrition, Chongqing, P.R. China; 3 Scientific Research Office, Ministry of Education Key Laboratory of Child Development and Disorders, National Clinical Research Center for Child Health and Disorders (Chongqing), China International Science and Technology Cooperation base of Child development and Critical Disorders, Children’s Hospital of Chongqing Medical University, Chongqing, P.R. China; 4 Department of Gastrointestinal Surgery and Neonatal Surgery, Ministry of Education Key Laboratory of Child Development and Disorders, National Clinical Research Center for Child Health and Disorders (Chongqing), China International Science and Technology Cooperation base of Child development and Critical Disorders, Children’s Hospital of Chongqing Medical University, Chongqing, P.R. China

**Keywords:** Gastrointestinal bleeding, Intestinal diseases

## Abstract

The fecal occult blood test (FOBT) is a screening tool for hematochezia. This study aims to summarize the clinical features associated with a positive FOBT in neonates and to explore some clues for the underlying causes. Combination with other clinical information, identifying the possible etiology is more likely and could be useful for choosing an effective therapeutic strategy. The medical records of 282 neonates with positive FOBTs from January 1 to July 31, 2016, were collected and retrospectively analyzed. The total incidence rate of FOBT positivity in neonates was 6.2%. Among these patients, 71 (25.2%) neonates had false-positive FOBTs, whereas 211 (74.8%) neonates had intraintestinal sources of hematochezia. Necrotizing enterocolitis (NEC, 20.9%), structural abnormalities of gastrointestinal tract (SAGT, 12.4%), and suspected food allergy (sFA, 10.6%) were the most common causes of neonatal hematochezia. It indicated that FOBT-positive neonates with NEC were more likely to suffer due to a younger gestational age, lower birth weight, and lower weight on admission than the neonates with other conditions. The proportions of neonates with bloody stool (90.0%) and diarrhea (63.3%) in the sFA group were markedly higher than those in the other groups. However, in the SAGT group, emesis (94.3%) and abdominal distension (80.0%) were evidently higher, usually accompanied by a relatively poor response (60.0%) and weakened bowel sounds (48.6%). Furthermore, the higher incidences of poor response (72.1%), abdominal distension (71.2%), bloody stools (64.4%), and weakened bowel sounds (62.7%) were observed in the NEC group. Due to the complicated etiology associated with a positive FOBT, the analyzed indexes were combined with other clinical features to identify the likely causes of neonatal hematochezia. Because NEC, sFA and SAGT show similar clinical manifestations and can occasionally transform into each other, close and frequent observation is crucial for timely intervention to achieve a better prognosis. Although it failed to provide an early warning of severe disease through FOBT, and the early intervention for FOBT might not decrease NEC, sFA, structural bowel injuries, or any other complications, newborn FOBT positive reminds medical staff to be alert to the related diseases including NEC, SAGT and sFA, by closer observation and follow-up.

## Introduction

Hematochezia, a common phenomenon among neonates, is defined as gastrointestinal bleeding due to a variety of causes involving digestive tract disorders and comorbidities of some critical illnesses, such as necrotizing enterocolitis (NEC), intestinal malrotation and volvulus, Hirschsprung disease, infectious colitis and systemic coagulopathy^1,2^.

The fecal occult blood test (FOBT), a screening test for hematochezia, is employed to detect invisible or hidden (occult) blood in the feces. It is simple and noninvasive. In adults, the FOBT is used as a screening tool for colorectal cancer associated with intestinal mucosal injury^3^. However, etiological data associated with positive FOBTs in neonates is scarce due to the particularity of the newborn population. It was reported that the positivity rate of FOBT in neonates is approximately 8.5%^4^, but it could be higher in extremely low birth weight neonates. Abramo *et al*. reported that the incidence rate of occult blood was 10% among neonates that were <1,800 g at birth^5^.

This study aimed to retrospectively analyze the characteristics associated with positive FOBTs in neonates admitted to our neonatal intensive care unit (NICU) by comparing the clinical characteristics and final diagnoses and attempting to explore some clues for improving efficiency of diagnosis and treatment.

## Results

### Underlying causes of positive FOBTs in neonates

Among the 282 neonates with positive FOBTs, 71 neonates (25.2%) had false-positive results, while the remaining 211 neonates (74.8%) had intraintestinal sources of bleeding. NEC, structural abnormalities of the gastrointestinal tract (SAGT) and suspected food allergy (sFA) were the most common causes of neonatal hematochezia and occurred in 59 neonates (20.9%), 35 neonates (12.4%), and 30 neonates (10.6%), respectively. Other etiologies included acute stress ulceration in 29 neonates (10.3%), acute diarrhea in 27 neonates (9.6%), postsurgical intestinal bleeding in 12 neonates (4.3%), functional abdominal distension in 10 neonates (3.5%), feeding intolerance in eight neonates (2.8%), and meconium constipation in one neonate (0.4%). False-positive FOBT results were caused by fecal contamination (often caused by pseudomenstruation and hematuresis) or other extraintestinal sources, including swallowed maternal blood (ingested during delivery or from split nipples) and neonatal blood (catheter-induced trauma to the upper airway or gastric mucosa).

### Clinical characteristics of neonates with NEC, SAGT and sFA

The clinical information for the first three etiologies in FOBT-positive neonates was analyzed. First, focus was directed to the onset time of the positive FOBT, and it was found that the median (IQR) onset times for NEC (7 [3, 13] days, data are presented as Median [P25, P75]), SAGT (10 [4, 17] days), and sFA (13 [8.75, 20.25] days) were significantly different (*X*^2^ = 9.318, *P* = 0.009). The gestational age (GA) (258 ± 20) days and birth weight (BW) (2.70 ± 0.64) kg in the SAGT group were significantly lower than those in the NEC group (272 ± 13) days and (3.17 ± 0.54) kg respectively, whereas in the sFA group (270 ± 12) days and (3.19 ± 0.42) kg, respectively (*P* < 0.001). In addition, the weight on admission in the NEC group (2.60 ± 0.64) kg was obviously lower than those in the SAGT and sFA groups (3.00 ± 0.53) kg and (3.13 ± 0.45) kg, respectively (*P* < 0.001, Table 1). The other observed items included gender, eutocia, gravity and parity, which did not have statistical differences (*P* > 0.05). These data indicated that neonates with NEC with positive FOBTs were more likely to suffer from a younger GA, lower BW, and lower weight on admission than those with other conditions.

**Table 1:** Demographic data of FOBT-positive neonates with NEC, SAGT and sFA.

Items		NEC	SAGT	sFA	*F*(*χ*^2^)	*P*
Male (%)		52.5 (31/59)	68.6 (24/35)	46.7 (14/30)	(3.579)	0.167
Eutocia (%)		38.9 (23/59)	34.3 (12/35)	26.7 (8/30)	(1.335)	0.513
Gravidity (%)	1	38.9 (23/59)	28.6 (10/35)	50.0 (15/30)	(4.818)	0.567
	2	25.4 (15/59)	34.3 (12/35)	16.7 (5/30)		
	3	16.9 (10/59)	17.1 (6/35)	10.0 (3/30)		
	≧4	18.7 (11/59)	20.0 (7/35)	23.3 (7/30)		
Parity (%)	1	71.2 (42/59)	48.6 (17/35)	56.7 (17/30)	(5.029)	0.078
	≧2	28.8 (17/59)	51.4 (18/35)	43.3 (13/30)		
GA (M ± SD, d)		258 ± 20	272 ± 13	270 ± 12	8.399	<0.001
BW (M ± SD, kg)		2.70 ± 0.64	3.17 ± 0.54	3.19 ± 0.42	11.233	<0.001
Weight on admission (M ± SD, kg)		2.60 ± 0.64	3.00 ± 0.53	3.13 ± 0.45	10.513	<0.001
Onset time [Median (P25~P75)] d		7 (3, 13)	10 (4, 17)	13 (8.75, 20.25)	(9.318)	0.009

GA: gestational age; BW: birth weight; M ± SD: mean ± standard deviation; d: day; Onset time: Onset time of positive FOBT; SAGT: structural abnormalities of the gastrointestinal tract.

### Risk factors and accompanying symptoms of neonates with NEC, SAGT and sFA

The differences in the delayed excretion of meconium, feeding patterns and the utilization of antibiotics among the three groups were statistically significant in terms of risk factors (*P* < 0.01). The proportion of neonates with delayed excretion of the meconium in the SAGT group was 37.1%, which was significantly higher than those in the other two groups (*P* < 0.01).The proportion of formula feeding and the administration of antibiotics before a positive FOBT in the NEC group was 61.0% and 52.5%, respectively, which were significantly higher than those in the other two groups (*P* < 0.01). The rates of accompanying symptoms, emesis, abdominal distension, diarrhea, bloody stool, weakened bowel sounds and poor response were all statistically significant among the three groups (*P* < 0.05). Furthermore, the proportions of neonates with bloody stool (90.0%) and diarrhea (63.3%) in the sFA group were markedly higher than those in the other groups. The rates of emesis (94.3%) and abdominal distension (80.0%) were evidently higher in the SAGT group than in the other groups, and these symptoms were accompanied by a relatively poor response (60.0%) and weakened bowel sounds (48.6%). Furthermore, high incidence rates of weakened bowel sounds (62.7%), poor response (72.1%), abdominal distension (71.2%), and bloody stool (64.4%) were observed in the NEC group (Table 2).

**Table 2:** Clinical characteristics of the FOBT-positive neonates with NEC, SAGT and sFA. NEC: necrotizing enterocolitis; SAGT: structural abnormalities of the gastrointestinal tract; sFA: suspected food allergy; CBC: complete blood count; AR: abdominal radiography.

Clinical characteristics			NEC (%)	SAGT (%)	sFA (%)	*F*(*χ*^2^)	*P*
Risk Factors	Gestational diabetes mellitus		11.9 (7/59)	5.7 (2/35)	3.3 (1/30)	(2.316)	0.314
	Meconium constipation		3.4 (2/59)	37.1 (13/35)	6.7 (2/30)	(20.770)	<0.001
	Premature rupture of membrane		23.7 (14/59)	11.4 (4/35)	16.7 (5/30)	(2.293)	0.318
	Meconium-stained amniotic fluid		18.6 (11/59)	14.3 (5/35)	6.7 (2/30)	(2.301)	0.316
	Patent ductus arteriosus		27.1 (16/59)	20.0 (7/35)	10.0 (3/30)	(3.545)	0.17
	Feeding manners	Formula	61.0 (36/59)	51.4 (18/35)	36.7 (11/30)	(21.450)	0.002
		Breast	8.5 (5/59)	14.3 (5/35)	30.0 (9/30)		
		Mixed	11.9 (7/59)	20.0 (7/35)	33.3 (10/30)		
		Other	18.6 (11/59)	14.3 (5/35)	0.0 (0/30)		
	Antibiotics application		52.5 (31/59)	34.3 (12/35)	13.3 (4/30)	(13.261)	0.001
Accompanying symptoms	Emesis		49.2 (29/59)	94.3 (33/35)	33.3 (10/30)	(28.318)	<0.001
	Abdominal distension		71.2 (42/59)	80.0 (28/35)	30.0 (9/30)	(20.188)	<0.001
	Diarrhea		27.1 (16/59)	11.4 (4/35)	63.3 (19/30)	(21.166)	<0.001
	Bloody stools		64.4 (38/59)	22.9 (8/35)	90.0 (27/30)	(31.501)	<0.001
	Weakened bowel sounds		62.7 (37/59)	48.6 (17/35)	23.3 (7/30)	(12.346)	0.002
	Fever		16.9 (10/59)	17.1 (6/35)	20.0 (6/30)	(0.139)	0.933
	Poor response		72.9(43/59)	60.0 (21/35)	23.3 (7/30)	(20.100)	<0.001
Auxiliary examinations	CBC	Neutrophils (%)	55.4 ± 16.9	57.1 ± 15.8	43.6 ± 12.0	6.882	0.001
		Neutrophils (*10^12^)	8.50 ± 7.70	8.42 ± 4.68	4.90 ± 2.25	3.789	0.025
		Neutrophil/lymphocyte (N/L)	2.28 ± 1.77	2.23 ± 1.64	1.12 ± 0.66	5.862	0.04
		Eosinophils (%)	7.6 ± 6.4	4.1 ± 4.0	7.8 ± 5.4	4.322	0.016
		Eosinophils (*10^12^)	0.76 ± 0.34	0.47 ± 0.18	0.79 ± 0.37	8.547	<0.001
		Platelet (*10^9^)	316 ± 135	311 ± 100	393 ± 159	3.788	0.025
	AR	Normal	1.8(1/57)	2.9 (1/34)	7.4 (2/27)	(33.983)	<0.001
		Mild	8.8(5/57)	44.1 (15/34)	14.8 (4/27)		
		Moderate	31.6(18/57)	32.4 (11/34)	48.1 (13/27)		
		Severe	47.4(27/57)	2.9 (1/34)	18.5 (5/27)		
		Reduction of bowel gas	10.5(6/57)	17.6 (6/34)	11.1 (3/27)		
Clinical outcomes	Improvement	Medical treatment	57.6(34/59)	14.3 (5/35)	86.7 (26/30)	(6.861)	0.032
		Surgical treatment	6.8(4/59)	68.6 (24/35)	0.0 (0/30)		
	Withdrawal from therapy		35.6(21/59)	17.1 (6/35)	13.3 (4/30)		

### Auxiliary examinations of the neonates with positive FOBTs due to NEC, SAGT and sFA

Complete blood count (CBC) indicated that the percentage of neutrophils was significantly increased in the NEC (55.4%) and SAGT (57.1%) groups (*P* < 0.001). Furthermore, the percentage of eosinophils in the NEC (7.6%) and sFA (7.8%) groups was obviously higher than those in SAGT group (4.1%) (*P* < 0.05). The platelet count in the sFA group was significantly elevated, reaching an average of 393 × 10^9^/L (*P* < 0.05), whereas the counts in the other two groups were 316 × 10^9^/L and 311 × 10^9^/L, respectively. The FOBTs in sFA neonates often showed elevated eosinophils and/or platelets in the CBC, which usually indicates vasculitis related to an allergic reaction, while the neonates in the NEC and SAGT groups often showed increased levels of neutrophils. Abdominal radiography revealed that the neonates in the NEC group mainly had a severe degree of bleeding (47.4%), whereas those in the sFA group (48.1%, moderate degree) appeared to have a slightly more severe degree than those in the SAGT group (44.1%, mild degree) (Tables 2 and 3).

**Table 3:** DAAS^25^ and degree of abnormal radiographic findings^26^.

Score	Finding	Degree of Severity
0	Normal gas pattern	Normal
1	Mild diffuse distention	
2	Moderate distention or normal with bubbly lucencies that are likely stool	Mild
3	Focal moderate distention of the bowel loop	
4	Separation or focal thickening of the bowel loops	
5	Featureless or multiple separated bowel loops	Moderate
6	Possible pneumatosis with other abnormal findings	
7	Fixed or persistent dilatation of the bowel loops	Severe
8	Pneumatosis is highly probable or definite	
9	Portal venous gas	
10	Pneumoperitoneum	

### Prognosis of the neonates with positive FOBTs due to NEC, SAGT and sFA

The prognoses included improvement and withdrawal from therapy. Improvement was defined as a marked alleviation. The neonates that showed improvement were further classified into the medical treatment group or the surgical & medical treatment group. In contrast, withdrawal from therapy was defined as discharged from the hospital too early because of serious complications or economic limitation.

It is clear that improvement was the main outcome in all three groups, accounting for more than 60.0% of the total. A total of 86.7% of sFA neonates, 57.6% of NEC neonates and 14.3% of SAGT neonates received medical treatment, whereas 68.6% of SAGT neonates and 6.8% of NEC neonates underwent surgical treatment. Moreover, 35.5% of NEC neonates, 17.1% of SAGT neonates, and 13.3% of sFA neonates were withdrawn from therapy. The differences among these groups were statistically significant (*X*^2^ = 6.861, *P* = 0.032; Table 2). In the NEC group, 21 neonates were withdrawn from therapy due to serious complications, including homeostasis disturbances, post-NEC intestinal stricture, and short bowel syndrome. However, 4 patients in the sFA group were discharged before complete enteral feeding due to economic factors or emotional factors such as unbearable being separated from their babies.

### Values of the clinical characteristics in the diagnosis differentiation of NEC, SAGT and sFA

To determine whether the statistically significant indexes in Table 2 were valuable for the differential diagnosis, the indexes were subjected to a multivariate analysis, including a discriminant analysis and multiple logistic regression analysis (Tables 2 and 4). The discriminant analysis indicated a correlation of BW (X_1_), delayed excretion of the meconium (X_2_), emesis (X_3_), diarrhea (X_4_), bloody stools (X_5_) and poor response (X_6_) with NEC (Y_NE_C = 0.010X_1_ + 1.515X_2_ + 3.641X_3_ − 1.201X_4_ + 4.029X_5_ + 6.761X_6_ − 19.433), SAGT (Y_SAGT_ = 0.012X_1_ + 4.291X_2_ + 6.002X_3_ − 1.619X_4_ + 2.644X_5_ + 6.595X_6_ − 25.853), and sFA (Y_sFA_ = 0.011X_1_ + 2.410X_2_ + 3.472X_3_ + 0.491X_4_ + 5.182X_5_ + 4.686X_6_ − 22.395). The maximum Y value was chosen, and the predicted probability was 71.0%, whereas the multivariate logistic regression analysis revealed a correlation of onset time (X_1_), antibiotic application, emesis (X_2_), and diarrhea (X_3_) with NEC (Y_NEC_ = −2.634 − 0.105X_1_ − 1.862X_2_ + 2.091X_3_) and SAGT (Y_SAGT_ = −10.654 − 0.125X_1_ − 2.59X_2_ + 2.651X_3_) when considering the sFA group as a reference. The maximum probability was chosen, and the predicted probability was 77.4%. The difference in the overall predicted probability between the two models was not statistically significant (*X*^2^ = 1.348, *P* = 0.246; Table 4).

**Table 4:** Discriminant analysis and multiple logistic regression analysis.

Group			Predicted etiology			total	Predicted probability (%)
			NEC	SAGT	sFA		
Actual etiology	Discriminant analysis	NEC	34	11	14	59	57.6
		SAGT	6	27	2	35	77.1
		sFA	1	2	27	30	90.0
		Total	41	40	43	124	71.0
	MLR analysis	NEC	48	3	8	59	81.4
		SAGT	7	26	2	35	74.3
		sFA	6	2	22	30	73.3
		Total	61	31	32	124	77.4

NEC: necrotizing enterocolitis; SAGT: structural abnormalities of the gastrointestinal tract; sFA: suspected food allergy; MLR: Multiple logistic regression.

## Discussion

Hematochezia is common during the neonatal period, manifesting as visible bloody stool or positive FOBT. The etiology is complicated, including the diseases of digestive tract and/or the complications of many critical disorders. Early identification of the cause is the key to its successful management. Due to the small amount of blood, some patients do not present visible blood in the stool, and a FOBT is needed to reduce misdiagnoses. Therefore, this study retrospectively analyzed the clinical characteristics of FOBT-positive neonates, providing a clinical experience for the etiological diagnoses and appropriate effective treatments. Our department was the largest NICU in western China. A total of 5,330 neonates were admitted from January 1 to July 31, 2016, including 1183 premature neonates and 4147 full-terms. Most of them suffered from perinatal asphyxia, severe pneumonia, sepsis, severe hemolysis and jaundice at or beyond the threshold level for exchange transfusion, prematurity and other critical illnesses. As a tertiary hospital affiliated of the medical university and the center of neonatal emergency-transportation system in western China, many neonates admitted to our department are transferred from out-of-town areas and are separated from their family, resulting in a relatively low rate of breastfeeding, approximately 30%, which is also a risk factor for intestinal damage, reflected as FOBT positivity. Therefore, the FOBT is employed as a routine screening test in our NICU. A number of conditions are related with positive FOBTs among neonates. Systemic problems related to hemorrhagic diseases or coagulopathy cause positive FOBTs. However, none of these disorders were found in this study, indicating that the incidence rate of occult hematochezia caused by systemic disorders in neonates is not as high as previously reported (1–2%)^6^. The present data revealed that the total incidence rate of FOBT positivity was 6.2%, and interestingly, 25.2% (71/282) of positive FOBTs in neonates were false-positives, which were caused by contaminated feces or the swallowing of maternal or neonatal blood. Furthermore, 74.8% (211/282) of bleeding was due to intestinal sources of hematochezia. Among these, NEC, SAGT and sFA were the most common causes, comprising 20.9%, 12.4% and 10.6% of the total positive FOBTs, respectively.

The onset time of NEC was inversely correlated with GA. NEC can present within the first week of life in term infants, whereas in preterm infants, it usually appears after commencement of feeds and occurs after the 2^nd^ and 3^rd^ weeks of life^7^. In this study, the average onset time of NEC in FOBT-positive infants was on the 7^th^ day after birth, which was probably related to preterm delivery in 35weeks of the 59 neonates with NEC. Furthermore, it was indicated that a neonate with a young GA, low rate of breastfeeding, exposure to antibiotics, and symptoms such as abdominal distention, weakened bowel sounds, or poor response should be considered to occurrence of NEC. In this situation, a CBC and abdominal radiography must be immediately carried out. Indeed, hematological abnormalities usually convey certain important and valuable prognostic information. Maheshwari *et al*.^8^ noted that increased neutrophil counts comprise an appropriate inflammatory response in patients with mild-moderately severe disease. In contrast, neutropenia can be seen in severe NEC. The present data showed that neonates in the NEC group had elevated neutrophil counts, which may be related with 64.4% [38/59] neonates in the NEC group diagnosed as stage I NEC. Additionally, Yang Y *et al*.^9^ reported that, neutrophil/lymphocyte (N/L) ratio could be good marker for the early diagnosis of NEC, distinguishing the severity. The data showed that neonates in the NEC group had elevated (N/L) ratio. Due to the small sample of NEC neonates, sub-group analysis was not performed, and the data was too limited to distinguish the severity. Large sample, multi-center studies are needed.

Traditionally, abdominal radiography has also played a crucial role in the diagnosis of NEC. Neonates with NEC often have severe intestinal lesions, which mainly reveal focal distention, fixed bowel loops, pneumatosis and portal venous gas on abdominal radiography. Furthermore, some of the neonates with NEC in this study presented with typical signs, categorized as moderate and severe intestinal injuries, on abdominal radiography (Tables 2 and 3). Wang *et al*.^10^ revealed that the recovery rate of NEC was 70–80%, whereas the improvement rate of NEC was 64.4% (38/59) in this study. Among the studied neonates, 34 received conservative treatment, four underwent surgery due to perforation or complete obstruction, and 21 were withdrawn from therapy due to serious complications, including homeostasis disturbances, post-NEC intestinal strictures, and short bowel syndrome. Gaudin *et al*.^11^ investigated the risk factors related to post-NEC intestinal stricture and indicated that the presence of peritonitis signs and thrombopenia were risk factors. Zhang *et al*.^12^ indicated that a late-onset time was a predictor of post-NEC intestinal stricture and that the elevation of white blood cells (WBCs) and procalcitonin (PCT) were observed in neonates with post-NEC intestinal stricture.

Previous studies have suggested that the incidence of sFA obviously increases after NEC or gastrointestinal tract surgery^13,14^. The symptoms of sFA are diverse and mainly occur from the 2^nd^ to 4^th^ week after contact with a food protein or other suspected substances. It was found that the onset time of a positive FOBT caused by sFA mostly appeared on the 2^nd^ week of life, and 93.3% (28/30) of the infants with GA more than 35 weeks. In addition, the incidence rates of gross bloody stools and diarrhea were 90.0% and 63.3%, respectively. Elevated eosinophil and platelet counts in the CBC appeared to indicate an allergic disorder, which was consistent with Feuille’s findings^15^. The signs on abdominal radiography mainly show diffuse distention and the separation or focal thickening of bowel loops. However, Kawai M *et al*.^16^ reported that the signs on abdominal radiography in infants with sFA presented recurrent episodes of pneumatosis-like NEC, which was difficult to differentiate from NEC. Similar radiographic signs were found in six neonates in this study. In terms of treatment, 86.7% (26/30) of the neonates improved after food avoidance, as observed in the present study. Suda *et al*.^17^ reported that some cases of sFA subsequently developed into NEC. They suggested that a persistent decrease in the platelet count may indicate this tendency. Therefore, dynamic follow-up of CBC should be performed to monitor progress timely.

Based on the present statistical data, it was found that Hirschsprung disease, intestinal stricture and intestinal atresia were frequent malformations in FOBT-positive neonates in the SAGT group, and the proportions were 37.1% (13/35), 14.3% (5/35), and 14.3% (5/35), respectively. Gosain *et al*.^18^ reported that neonates with Hirschsprung disease were at high risk for Hirschsprung-associated enterocolitis. The present data agree with this, as 13 neonates with positive FOBTs who had NEC-like clinical characteristics were finally confirmed to suffer from SAGT. Among these neonates, eight had Hirschsprung disease, four had intestinal stricture, and one had intestinal atresia. Of the SAGT neonates with positive FOBTs, 37.1% (13/35) had delayed excretion of the meconium, whereas 94.3% (33/35) had emesis. The CBC revealed elevated neutrophil counts, indicating the possibility of secondary infection. Since enteral structural abnormalities vary, different abdominal imaging characteristics contribute to diagnosis differentiation. Surgery can provide a definitive diagnosis but may not be suitable for repairing the intestinal structural abnormalities. Given this invasive approach, which may lead to postoperative complications such as adhesive intestinal obstruction, NEC and allergic enteritis, a precise grasp of the surgical opportunity is vital. In the present study, 68.6% (24/35) of the neonates with enteral structural abnormalities and positive FOBTs underwent surgery. Among these neonates, 5 neonates were readmitted for complications, 3 neonates had adhesive intestinal obstructions and two presented NEC within one year after the operation. Follow-up examinations were conducted on the readmitted patients. However, the data were still limited in reflecting the actual long-term prognosis.

**Figure 1:**
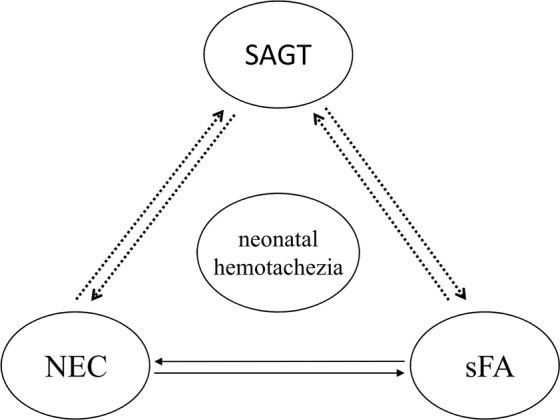
Common etiology of neonatal hematochezia.

SAGT occurs in neonatal period, including congenital and acquired. Congenital SAGT can be considered as malformations of gastro-intestinal tract. NEC, sFA and SAGT have the similar clinical manifestations, which sometimes transform into each other (Fig. 1). Congenital and acquired SAGT may cause intestinal obstruction and, consequently, local infection or inflammation due to damage to the intestinal barrier, resulting in a hypersensitivity to certain specific dietary antigens, manifesting as food allergy. If physicians encounter infants with surgical gastrointestinal disease, including intestinal malrotation, they need to consider food allergy in the differential diagnosis or as a complicating factor^13^. Stricture after NEC is a well-known common complication. After the acute episode of NEC, stricture, which is associated with severe and prolonged morbidity (septicemia, perforation, intestinal obstruction) and morbidity secondary to intestinal stricture, can develop during a variable period irrespective of the mode of management. Food allergy is a hypersensitive reaction to certain specific dietary antigens. Suda *et al*.^17^ reported that 2 neonates with sFA required laparotomy due to secondary NEC. Intestinal edema and injury would be induced when proper avoidance of suspected-food is not practiced, and severe intestinal inflammation might progress to local necrosis and perforation or endogenous infection, mimicking NEC and causing enteral complications such as intestinal stricture and other acquired SAGT. However, the treatments and prognoses of NEC, sFA and SAGT are usually different. Therefore, the investigators attempted to establish predictive models by discriminant analysis and multiple logistic regression analysis. Emesis and diarrhea are both involved as indexes in the two models, suggesting that these may have some clinical significance in the diagnosis. Unfortunately, no statistically significant difference was found in the overall predictive rate of these two models. Thus, no effective approach can be provided for the differential diagnosis at present. Hence, a prospective study is recommended to establish a valuable clinical prediction model.

**Figure 2:**
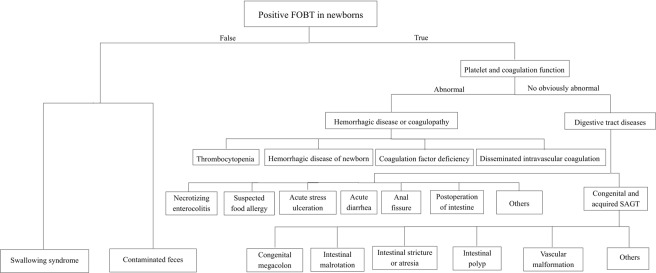
The diagnostic flow chart of positive FOBT in neonates.

In summary, for neonates with positive FOBTs, false-positive results should first be excluded. Then, systemic diseases and digestive tract disorders, such as infection, deformity, allergy, stress and injury, should be identified^1,19^ according to the process described in Fig. 2. NEC, SAGT and sFA were the most common causes of neonatal hematochezia. In newborns, those diseases show the similar clinical manifestations, and can occasionally transform into each other under some conditions. Pickering A *et al*.^20^ have reported that routine fecal occult blood testing does not predict necrotizing enterocolitis. Therefore, although we could not provide an early warning of severe disease though FOBT, and the early intervention for FOBT might not decrease NEC, sFA, structural bowel injuries, or any other complications, the significance of newborn FOBT positivity lies in that, medical staff would be alert to the related diseases including NEC, gastrointestinal malformations and suspected food allergy, with closer observation and follow-up. Based on the combination of the general condition, feeding tolerance, temperature, abdominal signs, CBC, routine stool tests plus FOBTs, and abdominal image examinations, clinicians would be able to identify the possible etiology, which is useful for timely modulating in the therapeutic strategy.

## Methods

### Study setting and patient selection

This study was conducted in the Neonatology Department of the Children’s Hospital of Chongqing Medical University. The data were collected from medical records of the Neonatology Department from January 1 to July 31, 2016. The study was approved by the Institutional Review Board of the Children’s Hospital of Chongqing Medical University. All methods were performed in accordance with the relevant guidelines and regulations. The data were collected, reviewed, deidentified, and analyzed anonymously by the authors; the ethics committee waived the requirement for informed consent because of the anonymized nature of the data and scientific purpose of the study.

### Subjects

A total of 5,330 neonates were screened. Among them, 1183 neonates were premature including 140 very low birthweight newborns. 330 neonates with positive FOBTs were preliminarily registered in the present study. The incidence rate of FOBT positivity was 6.2%. Two neonates with the incomplete medical records, and 46 neonates who had hematochezia after 28 days or whose GA ≥ 42weeks, were excluded. Thus, a total of 282 neonates were finally enrolled. The stool of individuals was tested at least once, 60 neonates were tested once and the others were tested twice or more. Among these subjects, 90 had a history of grossly visible bloody stools, and the FOBT was performed after the gross bloody stools disappeared.

### Data collection

Demographic, delivery and feeding history, physical examination (accompanying symptoms), treatment record, CBC and other specific examinations, including abdominal plain film examination (the most severe result was considered if there were multiple re-examinations), all data were collected from the Electronic Medical Record (EMR). NEC was defined based on Bell’s criteria with modifications^21^.

Adverse reactions associated with FA are frequent in the first year of life^22^. A challenge test with the causal food is indispensable in the diagnosis of food allergy but is not realistic for all cases in newborns^23^, because these tests may be dangerous and the results were difficult to analyze in neonates. Therefore, the concept of suspected food allergy might be more suitable for these cases. In our study, the diagnosis of sFA was based on a combination of some of the followings, a family history of anaphylactic diseases, a history of direct or indirect exposure to antibiotics and/or other suspected substances, and the exclusion of other possible causes of bloody stool. In addition, the symptoms were relieved with the feeding of amino acid milk powder or suspected-food avoidance, relapsing after the ingestion of the suspected food.

Acute stress ulcer is diagnosed according to preterm and term infants in the neonatal intensive care unit develop gastric mucosal lesions and present with upper GI bleeding suggested by gastric lavage fluid and have the exposure stress factors which includes perinatal asphyxia, intracranial hemorrhage, increased intracranial pressure, sepsis, hypoglycemia, severe vomiting, etc^24^.

Acute diarrhea was diagnosed according to the increased frequency and/or the changed characteristics of the stool compared to normal stool, whereas newborn diarrhea features the passage of loose or watery stools at least six times within 24 hours.

Structural abnormalities of the gastrointestinal tract (SAGT) were diagnosed based on biopsy or corresponding postoperative results, means “congenital or acquired SAGT”.

### Fecal occult blood test

FOBTs were performed at our clinical laboratory using the colloidal gold method (Aibo occult blood detection reagent; Aibo Biomedical, Hangzhou, China). Briefly, a stool sample was collected from the diaper. Then, a small amount of the collected fecal specimen was smeared in a test tube and immediately taken to the clinical laboratory. The assay was performed according to the product instructions. The result was considered positive when both the detection line and the quality control line appeared.

### Evaluation of abdominal radiography

Abdominal radiography was evaluated based on the Duke Abdominal Assessment Scale (DAAS)^25^. Since reduced intestinal inflammation is not included in the DAAS, this was analyzed separately. The degree of severity referred to the study by Boyin Deng *et al*.^26^ (Table 3).

### Statistical analysis

All data were analyzed using *SPSS* 17.0 software (Chicago, IL, USA). Normally distributed continuous data were described as the mean ± standard deviation (M ± SD) and examined by analysis of variance. Skewed data were described as the median (interquartile range [IQR]) and analyzed by the *Kruskal-Wallis* test. Categorical data were analyzed by the *chi-square* test or *Fisher’s* exact test. A multivariate analysis, including discriminant and multiple logistic regression analyses, was applied to analyze the differential diagnosis. A *P*-value < 0.05 was considered statistically significant.

### Ethics approval and consent to participate

The study protocol was approved by the Institutional Review Board of the Children’s Hospital of Chongqing Medical University.

### Informed consent statement

This is a retrospective study. The ethics committee waived the requirement for informed consent because of the anonymized nature of the data and scientific purpose of the study.
